# Informal Human Milk Sharing Among US Mothers

**DOI:** 10.1001/jamanetworkopen.2025.42036

**Published:** 2025-11-06

**Authors:** Jill R. Demirci, Molly Waymouth, Kristin N. Ray, Kortney Floyd James, Lori Uscher-Pines

**Affiliations:** 1 Department of Health Promotion and Development, University of Pittsburgh School of Nursing, Pittsburgh, Pennsylvania; 2 RAND Corporation, Arlington, Virginia; 3 School of Medicine, University of Pittsburgh, Pittsburgh, Pennsylvania; 4 RAND Corporation, Santa Monica, California

## Abstract

This survey study evaluates reported use of human milk acquired from informal sources in a diverse, national US sample.

## Introduction

Informal milk sharing—noncommodified exchange of human milk, often via online or personal networks^1^—is a controversial practice. Recipient families are typically highly attuned to breastfeeding benefits and milk-sharing risks and do not qualify for pasteurized donor human milk (PDHM) from accredited milk banks.^1^ A 2013 survey found 29% of mothers (majority North American) with low supply used shared milk,^2^ but current US population-level prevalence remains unknown. This analysis explored reported use of human milk acquired from informal sources in a diverse, national sample.

## Methods

Data were drawn from a randomized clinical trial of telelactation that recruited nulliparous pregnant women (33-37 weeks) intending to breastfeed across 39 US states from July 2021 to December 2022. Participants were recruited through parenting app advertisements.^3^ Ethical approval was granted by the RAND institutional review board, and participants provided written informed consent in the original study. American Association for Public Opinion Research (AAPOR) reporting guidelines were followed.

Participants completed web-based surveys at enrollment and postpartum weeks 4 and 24. At 24 weeks, participants were queried whether they ever fed their infant donor breast milk from a milk bank (PDHM), breast milk from a milk sharing network, and/or breast milk from friends or family. The latter 2 categories were collapsed into a shared milk category. Prenatal breastfeeding beliefs and postnatal breastfeeding perceived success were measured via validated Likert-scale items drawn from the Infant Feeding Practices-II survey (eAppendix in Supplement 1). Data were analyzed descriptively. Associations between donor or shared milk use, participant characteristics, and breastfeeding outcomes were examined with Pearson χ^2^ or Fisher exact tests (categorical) and independent sample *t* tests (continuous), comparing users with nonusers. A 2-sided *P* < .05 was considered significant. Prevalence of donor or shared milk use is reported with Clopper–Pearson 95% binomial CIs. Analyses were completed in SPSS software versions 30 and 31 (IBM) from February to October 2025.

## Results

Donor or shared milk use data were available for 1909 of 2108 enrolled participants (mean [SD] maternal age, 29.6 [5.4] years). Participants were demographically diverse, with 610 (32%) African American or Black, 58 (3%) American Indian or Alaska Native, 97 (5.1%) Asian, and 983 (51.5%) White participants (Table 1). At 24 weeks post partum, 180 participants (9.4%) reported feeding their infant donor or shared milk. The most common source was PDHM, (117 participants [6.1%]), followed by shared milk (70 participants [3.7%]), including using milk from friends or family (52 participants [2.7%]), milk-sharing networks (17 participants [0.9%]), and other sources (6 participants [0.3%]). Ten (0.5%) used both PDHM and shared milk.

**Table 1.** Sample Characteristics by Use of Any Nonmaternal Human Milk Sources Within 6 Months After Birth

Characteristic	Total participants, No. (%) (N = 1909)	Shared milk		PDHM		Shared and/or PDHM	
		Participants, No. (%) (n = 70)	*P* value^a^	Participants, No. (%) (n = 117)	*P* value^a^	Participants, No. (%) (n = 180)	*P* value^a^
Maternal age, mean (SD)^b^	29.6 (5.4)	29.2 (5.4)	.56	30.3 (5.2)	.15	29.9 (5.4)	.51
Hispanic^b^	747 (39.2)	28 (40)	.90	34 (29.1)	.02	58 (32.2)	.05
Race^b^^,^^c^							
African American or Black	610 (32)	16 (22.9)	.12	40 (34.2)	.61	55 (30.6)	.68
American Indian or Alaska Native	58 (3)	4 (5.7)	.16	4 (3.4)	.78	7 (3.9)	.49
Asian	97 (5.1)	3 (4.3)	>.99	6 (5.1)	>.99	9 (5)	>.99
White	983 (51.5)	40 (57.1)	.39	64 (54.7)	.51	100 (55.6)	.27
Education							
High school or less	244 (12.8)	11 (15.7)	.76	16 (13.7)	.43	26 (14.4)	.66
Associate’s degree or some college	557 (29.2)	20 (28.6)		28 (23.9)		48 (26.7)	
Bachelor’s degree or higher	1108 (58)	39 (55.7)		73 (62.4)		106 (58.9)	
Marital status							
Married	1142 (59.8)	46 (65.7)	.06	79 (67.5)	.09	118 (65.6)	.25
Partnered	570 (29.9)	13 (18.6)		32 (27.4)		45 (25)	
Single	197 (10.3)	11 (15.7)		6 (5.1)		17 (9.4)	
Insurance during pregnancy^d^							
Private or commercial	1314 (68.8)	45 (64.3)	.43	94 (80.3)	.01	135 (75)	.06
Medicare and/or Medicaid	605 (31.7)	23 (32.9)	.83	26 (22.2)	.02	47 (26.1)	.09
Military	69 (3.6)	1 (1.4)	.52	4 (3.4)	>.99	4 (2.2)	.31
None	23 (1.2)	4 (5.7)	.01	0	.40	4 (2.2)	.27
Household income per y							
<$40 000	503 (26.3)	25 (35.7)	.27	15 (12.8)	.002	38 (21.1)	.24
$40 000 to <$80 000	476 (24.9)	13 (18.6)		38 (32.5)		49 (27.2)	
>$80 000	787 (41.2)	28 (40)		50 (42.7)		75 (41.7)	
Don’t know or unsure	143 (7.5)	4 (5.7)		14 (12)		18 (10)	
Community of residence							
Urban	646 (33.8)	24 (34.3)	.65	37 (31.6)	.79	56 (31.1)	.71
Suburban	1166 (61.1)	41 (58.6)		75 (64.1)		114 (63.3)	
Rural	97 (5.1)	5 (7.1)		5 (4.3)		10 (5.6)	
Speak a language(s) other than English at home	550 (28.8)	20 (28.6)	>.99	26 (22.2)	.11	45 (25)	.26
NICU admission^b^^,^^e^	200 (10.9)	4 (6.0)	.23	30 (26.3)	<.001	33 (19)	<.001
Infant gestational weeks at birth, mean (SD) [range]^b^^,^^e^	39.0 (1.3) [33-41]	38.9 (1.2)	.52	38.5 (1.6)	.002	38.6 (1.5)	.002

Abbreviations: NICU, neonatal intensive care unit; PDHM, pasteurized donor human milk.

^a^ Assessed via maternal survey at baseline (33-37 weeks pregnant) unless otherwise noted. Values significant at α < .05; *P* values for categorical variables derived from Pearson χ^2^ tests, with Fisher exact tests used when cell counts were less than expected; *P* value for continuous variable of infant gestational age derived from independent samples *t* test; *P* values reflect comparisons between users and nonusers of the nonmaternal human milk source for each characteristic.

^b^ Data available for fewer participants than sample total: 1908 for maternal age and Hispanic ethnicity, race, 1833 for NICU admission, and 1834 for infant gestation.

^c^ Race categories not mutually exclusive; participants could choose more than 1 race from 7 different categories, including some other race; Middle Eastern or North African; Native Hawaiian, Samoan, Chamorro, other Pacific Islander, and some other race not represented, as each category made up 1% or less of sample.

^d^ Indian Health Service coverage not tabulated due to low cell count (2 of 1909 participants); participants could report multiple forms of insurance.

^e^ NICU admission and infant gestational age assessed at postpartum week 4 survey; survey asked participants to round down to the nearest completed week of pregnancy (eg, 37 weeks, 6 days pregnant = 37 weeks).

Few demographic differences were observed between users and nonusers of shared milk, although shared milk use was more likely among uninsured participants (odds ratio, 5.8; 95% CI, 1.9-17.5; *P* = .01). PDHM and shared milk users had strong prenatal commitment to breastfeeding, with the strongest beliefs about meeting breastfeeding goals among shared milk users. However, participants who used either PDHM or shared milk were less likely than nonusers to feel successful at breastfeeding at 24 weeks (χ^2^_4_ = 10.0; *P* = .04). There were no significant differences in infant formula use at 24 weeks post partum among PDHM (χ^2^_1_ = 0.27; *P* = .61), shared milk (χ^2^_1_ = 1.02; *P* = .31), and PDHM or shared milk (χ^2^_1_ = 0.05; *P* = .82) (Table 2).

**Table 2.** Maternal Breastfeeding Beliefs During Pregnancy (33-37 Weeks’ Gestation) and Breastfeeding Outcomes at 6 Months Post Partum by Use of Any Nonmaternal Human Milk Sources

Characteristic	Total participants, No. (%) (N = 1909)	Shared milk		PDHM		Shared and/or PDHM	
		Participants, No. (%) (n = 70)	*P* value^a^	Participants, No. (%) (n = 117)	*P* value^a^	Participants, No. (%) (n = 180)	*P* value^a^
Breastfeeding beliefs prenatally							
Opinion on best way to feed a baby							
Breast milk only	1690 (88.5)	63 (90)	.76	111 (94.9)	.02	166 (92.2)	.12
Formula only	0	0		0		0	
Mix of breast milk and formula	195 (10.2)	6 (8.6)		4 (3.4)		11 (6.1)	
No opinion	24 (1.3)	1 (1.4)		2 (1.7)		3 (1.7)	
Feeding plan (first mo post partum)^b^							
Breast milk only	1708 (89.5)	61 (87.1)	.55	108 (92.3)	.35	163 (90.6)	.70
Formula only	0	0		0		0	
Breast milk and formula	200 (10.5)	9 (12.9)		9 (7.7)		17 (9.4)	
Don’t know yet/not sure	0	0		0		0	
I believe I will be able to meet my breastfeeding goals							
Strongly agree	758 (39.7)	33 (47.1)	.01	48 (41)	.43	76 (42.2)	.03
Agree	946 (49.6)	25 (35.7)		53 (45.3)		77 (42.8)	
Neither agree nor disagree	196 (10.3)	10 (14.3)		15 (12.8)		24 (13.3)	
Disagree	9 (0.5)	2 (0.4)		1 (0.9)		3 (1.7)	
Strongly disagree	0	0		0		0	
Breastfeeding outcomes and perceived success at 6 mo post partum							
Any commercial infant formula use past 24 h^b^^,^^c^	1089 (57)	44 (62.9)	.31	64 (54.7)	.61	104 (57.8)	.82
In general, I am/was satisfied with breastfeeding^b^^,^^c^							
Strongly agree	714 (38.6)	20 (29.4)	.19	37 (32.5)	.34	55 (31.4)	.18
Agree	541 (29.2)	20 (29.4)		34 (29.8)		52 (29.7)	
Neutral	208 (11.2)	11 (16.2)		11 (9.6)		22 (12.6)	
Disagree	266 (14.4)	9 (13.2)		21 (18.4)		29 (16.6)	
Strongly disagree	123 (6.6)	8 (11.8)		11 (9.6)		17 (9.7)	
In general, I feel/felt successful at breastfeeding my baby^b^^,^^c^							
Strongly agree	743 (40.1)	18 (26.5)	.06	38 (33.3)	.17	56 (32.0)	.04
Agree	442 (23.9)	16 (23.5)		28 (24.6)		41 (23.4)	
Neutral	167 (9.0)	6 (8.8)		10 (8.8)		16 (9.1)	
Disagree	260 (14.0)	13 (19.1)		15 (13.2)		28 (16.0)	
Strongly disagree	240 (13.0)	15 (22.1)		23 (20.2)		34 (19.4)	
How likely is it that you would breastfeed if you had another child?							
Very likely	1295 (67.8)	42 (60)	.38	81 (69.2)	.37	119 (66.1)	.31
Likely	347 (18.2)	15 (21.4)		21 (17.9)		35 (19.4)	
Neutral	145 (7.6)	9 (12.9)		12 (10.3)		19 (10.6)	
Unlikely	70 (3.7)	2 (2.9)		1 (0.9)		3 (1.7)	
Very unlikely	52 (2.7)	2 (2.9)		2 (1.7)		4 (2.2)	

Abbreviation: PDHM, pasteurized donor human milk.

^a^ Significant at α < .05; *P* values derived from Pearson χ^2^ tests, with Fisher exact tests used when cell counts were less than expected; *P* values reflect comparisons between users and nonusers of the nonmaternal human milk source for each characteristic.

^b^ Data available for fewer participants than sample total; 1908 for feeding plan, 1912 for commercial formula use past 24 hours, and 1852 for satisfied with breastfeeding and felt successful breastfeeding.

^c^ For those who stopped breastfeeding, asked in past tense; for those still breastfeeding, asked in present tense (only asked for those who initiated breastfeeding).

## Discussion

In this large, diverse sample of first-time mothers in the US, 1 in 27 participants reported feeding their infants shared human milk. Prior work indicates that families engage in many, but not all, recommended milk sharing risk mitigation measures^1^ and fear disclosing their milk sharing to practitioners.^4^ Given the prevalence in our sample, pediatric practitioners should be aware that milk sharing occurs across demographics; they can counsel families considering or using shared milk on risks and risk-reduction strategies recommended by the Academy of Breastfeeding Medicine^5^ and the American Academy of Nursing,^6^ while noting that evidence on home pasteurization is still evolving.

Participants using either PDHM or shared milk were more likely to feel unsuccessful at breastfeeding and used infant formula at comparable rates with nonusers, despite assumptions that donor milk reduces formula use. Thus, lactation support remains critical, even when alternate sources of human milk are available. Limitations include data that were self-reported and not drawn from a nationally representative source and that there was no assessment of motives, volume, or timing of donor or shared milk use.

## Notes

Supplement 1. eAppendix. Survey Item Wording

Supplement 2. Data Sharing Statement
