# Supplementary material for: Informal Human Milk Sharing Among US Mothers
Source: JAMA Netw Open. 2025 Nov 6;8(11):e2542036. doi: 10.1001/jamanetworkopen.2025.42036 (PMC12593121; doi:10.1001/jamanetworkopen.2025.42036)
Supplement: Supplement 1. — eAppendix. Survey Item Wording [file jamanetwopen-e2542036-s001.pdf]

Supplemental Online Content

Demirci JR, Waymouth M, Ray KN, James KF, Uscher-Pines L. Informal human milk sharing among US mothers. *JAMA Netw Open*. 2025;8(11):e2542036. doi:10.1001/jamanetworkopen.2025.42036

eAppendix. Survey Item Wording

This supplemental material has been provided by the authors to give readers additional information about their work.

© 2025 Demirci JR et al. *JAMA Network Open*.

Supplement: Survey Item Wording

Non-Maternal Milk Sources Items (Assessed at 24-Week Postpartum Survey)

1. Has your baby ever been fed another person's breast milk (e.g., donor milk, shared milk)?
    a. Yes
    b. No

2. [if item 1 answered YES] Not including your own breast milk, what types of breast milk has your baby been fed? Check ALL that apply.
    a. Donor breast milk from a milk bank
    b. Breast milk from a milk sharing network (e.g., Human Milk 4 Human Babies)
    c. Breast milk from a friend or family member
    d. Other, please describe
    e. I don't know

Demographics/Sample Characteristic Items (Assessed at Enrollment/Baseline Survey at 33-37 Weeks of Pregnancy)

1. How old are you? (populated scrollable list for participant selection of integer)

2. Are you of Hispanic, Latino, or Spanish origin? Check ALL that apply.
    a. No, not of Hispanic, Latino, or Spanish origin
    b. Yes, Mexican, Mexican American, Chicano
    c. Yes, Puerto Rican
    d. Yes, Cuban
    e. Yes, another Hispanic, Latino, or Spanish origin (for example, Salvadoran, Dominican, Colombian, Guatemalan, Ecuadorian)

3. What is your race? Check ALL that apply.
    a. Black or African American (for example, African American, Nigerian, Haitian)
    b. Middle Eastern or North African (for example, Ethiopian, Lebanese, Egyptian)
    c. American Indian or Alaskan Native (for example, Navajo Tribe, Blackfeet Tribe)
    d. White (for example, German, Irish, English, Italian)
    e. Asian (Chinese, Filipino, Vietnamese, Korean, Japanese, Asian Indian)
    f. Native Hawaiian, Samoan, Chamorro, other Pacific Islander
    g. Some other race

© 2025 Demirci JR et al. *JAMA Network Open*.

4. What is the highest degree or level of school you COMPLETED?
    a. Grade school
    b. Some high school
    c. High school graduate or GED
    d. Some college, no degree (1-3 years)
    e. Associate degree in college (2 years)
    f. Bachelor's degree (e.g. BA, AB, BS)
    g. Graduate degree

5. What is your current marital status? Please check one.
    a. Married
    b. Living with partner, not married
    c. Living apart from partner, not married
    d. Single
    e. Widowed
    f. Divorced
    g. Separated

6. During your current pregnancy, what kind of health insurance do you have to pay for your prenatal care? Check ALL that apply.
    a. Private health insurance (for example, through an employer or union or through your state's health exchange)
    b. Medicaid (health insurance program run by your state that provides coverage for lower-income people)
    c. Medicare (federal health insurance program for people who are 65 or older and for certain younger people with disabilities)
    d. Military health care (TRICARE/VA/CHAMPVA)
    e. Indian Health Service
    f. I have not had any health insurance coverage during my current pregnancy.

7. Over the last 12 months, what was your total yearly household income before taxes? Include your income, your partner's income, and any other income you may have received such as alimony, child support, and financial assistance from the state or federal government. All information will be kept private. Would you say it was between…
    a. $0 to $14,999
    b. $15,000 to $24,999
    c. $25,000 to $39,999
    d. $40,000 to $54,999
    e. $55,000 to $79,999
    f. $80,000 or more
    g. I don't know/not sure

© 2025 Demirci JR et al. *JAMA Network Open*.

8. Which of the following best describes the community you live in now?
    a. A large city
    b. A suburb near a large city
    c. A small city or town
    d. A rural area

9. Do you speak a language other than English at home?
    a. Yes
    b. No

NICU & Gestational Age Items (Assessed at 4-Week Postpartum Survey)

1. Did your baby have to stay in an intensive care unit (NICU) at any time after birth?
    a. Yes
    b. No

2. How many weeks pregnant were you when your baby was born? (Please round down when you answer. For example, if you were 37 weeks and 6 days pregnant, check 37 weeks.)
    a. 33 weeks
    b. 34 weeks
    c. 35 weeks
    d. 36 weeks
    e. 37 weeks
    f. 38 weeks
    g. 39 weeks
    h. 40 weeks
    i. 41 or more (overdue)

Beliefs about Breastfeeding Assessed at Enrollment/Baseline Survey at 33-37 Weeks of Pregnancy

1. Which of the following is closest to your opinion? The best way for me to feed my baby is to give:
    a. Breast milk only (breastfeeding and/or pumped breast milk)
    b. Formula only
    c. A mix of both breast milk and formula
    d. No opinion

2. How do you plan to feed your new baby in the first month after birth?
    a. Breast milk only (my baby will not be given formula)
    b. Formula only (none of my own milk)
    c. Both breast milk and formula
    d. I don't know yet/not sure

© 2025 Demirci JR et al. *JAMA Network Open.*

3. I believe I will be able to meet my breastfeeding goals.
    a. Strongly agree
    b. Agree
    c. Neither agree nor disagree
    d. Disagree
    e. Strongly disagree

Beliefs about Breastfeeding and Breastfeeding Outcomes Assessed at 24-Week Postpartum Survey

1. In the last 24 hours, how much formula was your baby fed? Please give your best guess.
    a. None: my baby was not fed any formula in the last 24 hours
    b. Less than 7 ounces
    c. 7-12 ounces
    d. 13-22 ounces
    e. 23 or more ounces

2. In general, I [am/was] satisfied with breastfeeding. *Note: tense modified depending on whether still breastfeeding*
    a. Strongly Disagree
    b. Disagree
    c. Neither agree nor disagree
    d. Agree
    e. Strongly Agree

3. In general, I [feel/felt] successful at breastfeeding my baby. *Note: tense modified depending on whether still breastfeeding*
    a. Strongly Disagree
    b. Disagree
    c. Neither agree nor disagree
    d. Agree
    e. Strongly Agree

4. How likely is it that you would breastfeed if you had another child?
    a. Very unlikely
    b. Unlikely
    c. Neutral
    d. Likely
    e. Very likely

© 2025 Demirci JR et al. *JAMA Network Open*.
