# Supplementary material for: Informal Human Milk Sharing Among US Mothers
Source: JAMA Netw Open. 2025 Nov 6;8(11):e2542036. doi: 10.1001/jamanetworkopen.2025.42036 (PMC12593121; doi:10.1001/jamanetworkopen.2025.42036)
Supplement: Supplement 2. — Data Sharing Statement [file jamanetwopen-e2542036-s002.pdf]

Data Sharing Statement

Demirci. Informal Human Milk Sharing. *JAMA Netw Open*. Published November 06, 2025.
doi:10.1001/jamanetworkopen.2025.42036

Data
Data available: Yes
Data types: Deidentified participant data
How to access data: Email: Lori Uscher-Pines, luscherp@rand.org
When available: With publication

Supporting Documents
Document types: None

Additional Information
Who can access the data: Researchers whose proposed use of the data has been approved (data sharing agreement).
Types of analyses: for any purpose
Mechanisms of data availability: signed data access agreement
